# A Rare Phenomenon of Lithium-Associated Acne Inversa: A Case Series and Literature Review

**DOI:** 10.7759/cureus.36051

**Published:** 2023-03-12

**Authors:** Dhananjay Chaudhari, Rimsha R Vohra, Munira Abdefatah Ali, Huzaifa Nadeem, Baris Tarimci, Tulika Garg, Rawan A Sharari, Abia Joseph, Aadil Khan

**Affiliations:** 1 Department of Psychiatry, Ganesh Shankar Vidyarthi Memorial (GSVM) Medical College and Lala Lajpat Rai (LLR) Hospital, Kanpur, IND; 2 Internal Medicine, Dow University of Health Sciences, Karachi, PAK; 3 College of Medicine, University of Illinois at Chicago, Chicago, USA; 4 Psychiatry, Combined Military Hospital (CMH) Lahore Medical College, Lahore, PAK; 5 Internal Medicine, Ege University Faculty of Medicine, Izmir, TUR; 6 Medicine, Government Medical College and Hospital, Chandigarh, Chandigarh, IND; 7 Family Medicine, Washington University of Health and Science, Montreal, CAN; 8 Surgery, John F. Kennedy University School of Medicine, Willemstad, CUW; 9 Department of Internal Medicine, Lala Lajpat Rai (LLR) Hospital, Kanpur, IND

**Keywords:** hypoxia-induced factor-1, neutrophilia, bipolar affective disorder, acne inversa, lithium

## Abstract

Lithium use has been associated with dermatological issues, including psoriasis, folliculitis, and acneiform outbreaks. The lithium dosage and the therapeutic range of serum lithium levels are closely correlated with the frequency of cutaneous adverse effects. Lithium-induced acne inversa is a less well-known adverse effect, causing significant morbidity. Acne inversa (hidradenitis suppurativa) is a chronic inflammatory illness of the skin seen in the folds of the skin and face and distinguished by the presence of painful nodules and fistulas, as well as a propensity for tissue fibrosis. We report two cases of bipolar affective disorder who received long-term lithium treatment and experienced acne inversa during treatment, which subsided once the lithium was withdrawn.

## Introduction

Lithium is still considered the primary mode of treatment for bipolar disorders despite the availability of multiple new therapies [1]. Various studies have been done to understand the effects of lithium in psychiatric illness management; however, the mechanism of action beyond its mood-stabilizing impacts still needs to be better understood [2]. Lithium works as a sodium transport modifier at the muscle and nerve cell level, while at the intracellular level, lithium works on the second-messenger systems (cAMP) function modification. These mechanisms eventually alter specific neurotransmitters' metabolism and neurotransmission [3].

However, safety concerns throughout the treatment remain significant due to its narrow therapeutic window and major side effects like hypothyroidism and cardiac and renal dysfunction; thus, it requires monitoring. Common side effects include gastrointestinal symptoms like nausea, diarrhea, urinary system problems like increased urinary frequency, and excessive thirst [4]. Weight gain and impaired cognition tend to be more stressful to the patients and may contribute to poor medication adherence [5].

Lithium-associated dermatological disorders are another unfavorable side effect with an approximate prevalence of 45% [6]. The most recognizable cutaneous presentations are the progression of previously existing or newly diagnosed psoriasis, alopecia, acne, follicular inflammation, and maculopapular rashes [7]. Acneiform disorders will likely arise during the first six months of the treatment; some studies have demonstrated lithium's role in enhancing the circulating neutrophil chemotaxis process. Subsequently, lysosomal enzymes are released, essential in follicular hyperkeratosis and thus the formation or aggravation of previously formed acne [8]. The usual clinical presentation of these cases is the formation of monomorphic papules or pustular lesions, sometimes complicated by comedone or cyst formation. These lesions are typically in the trunk and body extremities [9]. We present two cases of acne inversa after using lithium medication for different psychiatric diseases.

## Case presentation

Case 1

**Figure 1:**
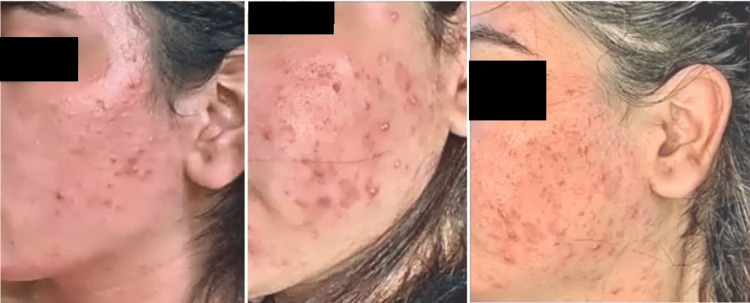
Multiple severe cystic eruptions, papules, nodules, and comedones with some sinus tracts and scarring.

A 26-year-old female with a history of bipolar affective disorder (BPAD) and substance abuse of nicotine presented with symptoms of depression, owing to which antidepressants were prescribed. However, on follow-up after two weeks, she developed mania, following which the medication regimen was shifted to lithium 300 mg twice a day. After a month follow-up, she started suffering from severe cystic eruptions, followed by papules, nodules, and comedones on her face and neck. There were no lesions on other parts of the body. She reported no further history of dermatologic disease of any kind. She was referred to a dermatologist for an opinion. On further evaluation, the results of her serum chemistry and complete blood count were within the normal range. Serum lithium and sex hormone levels were also within normal range. A diagnosis of acne inversa (Hurley stage II) was made due to the presence of multiple bumps with some sinus tracts and scarring caused by lithium therapy (Figure 1). She was prescribed doxycycline and Neosporin for local application. However, she didn't show much improvement to the treatment given, and it was assumed that lithium was causing adverse drug reactions and causing acne inversa, owing to which her treatment was shifted to oxcarbazepine 150mg twice a day. Lithium was discontinued, and she was discharged with a follow-up after four weeks. On her recent follow-up, she reported improvement in her skin condition.

Case 2

**Figure 2:**
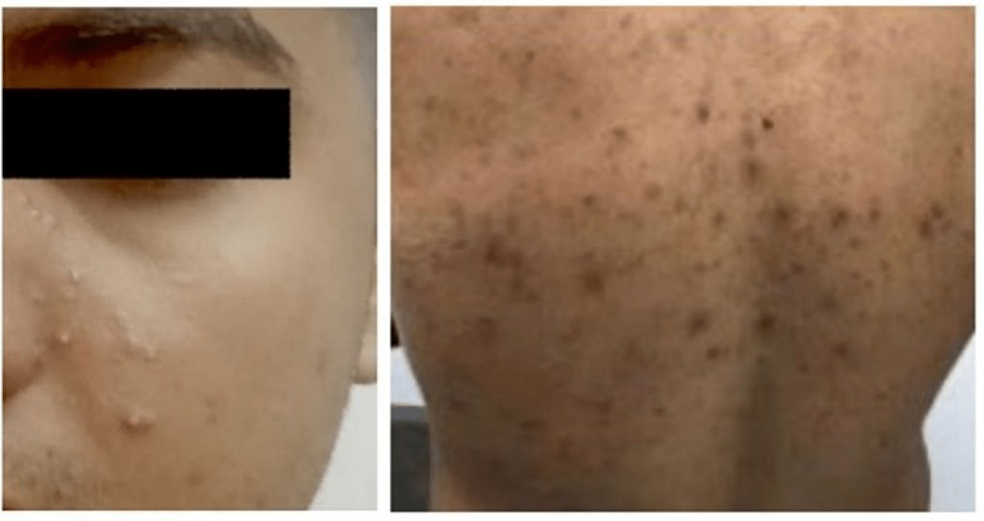
Multiple bumps, including nodules and comedones with some sinus tracts and scarring.

A 31-year-old male presented with a chief complaint of depression, and his past medical history is significant for hypertension, hyperlipidemia, and hyperuricemia. On further exploration, it was revealed that he was a hyperthymic personality. He also had a history of alcohol abuse, cocaine, and nicotine. He was put on antidepressants venlafaxine 375mg daily. He showed a slight improvement in the treatment given. However, active suicidal thought was present, for which lithium 900mg daily was added. Significant progress was observed. However, on follow-up after one month, nodulocystic acne was noticed in the trunk, full back, and lower abdomen, as shown in Figure 2. The patient went to the dermatologist and was diagnosed with acne inversa. He had no history of acne or dermatologic disease. On further evaluation, the results of his serum chemistry and complete blood count were within the normal range. Serum lithium level was also within normal range.

He was prescribed doxycycline and local applicants; however, no significant improvement was seen within 14 days. However, his suicidal thought improved. A diagnosis of lithium-induced acne inversa was made, and he was switched to carbamazepine and venlafaxine. Withdrawal of lithium resulted in improvement of his skin condition.

## Discussion

The chemical lithium, which psychiatrists prescribe to treat bipolar affective disorders, has the highest rate of cutaneous side effects of any psychoactive substance [1]. The most frequent cutaneous responses to lithium include acneiform eruptions, psoriasis, maculopapular eruptions, and follicular eruptions [2-4]. The exact mechanism by which lithium causes these reactions is yet not known. Acne inversa is an uncommon side effect of lithium medication and is not widely reported in the literature. We have tabulated reported cases of lithium-induced severe adverse skin reactions in Table 1 [10-13].

**Table 1:** Reported cases of lithium-induced severe induced skin reactions. M: male, F: female, AE: adverse event.

Authors	Age/Sex	Diagnosis	Lithium treatment duration (days)	Cutaneous AEs	Response to lithium discontinuation/dose reduction
Bugueno JM et al. [10]	25/F	Bipolar disorder	29	Lichenoid lesion, nodulocystic acne	Yes
Sacrfi F et al. [11]	40/F	Bipolar disorder	118	Fascial acne, acne inversa	Yes
Wang EH et al. [12]	37/F	Bipolar disorder	7	Skin lesions all over body, acne inversa	Yes
Meijima H et al. [13]	55/M	Psychosis	Not reported	Psoriasis, nodulocystic acne, acne inversa	Yes

Patients using lithium frequently experience the onset or flare-up of acneiform lesions. The pathogens causing the two illnesses might be identical. Initially, it was believed that hidradenitis suppurativa mainly affected the apocrine glands. However, further histopathologic findings have shown that apocrine gland involvement might range from modest to absent in some cases [7]. It has been hypothesized that lithium may cause significant early alterations in acne inversa, including follicular blockage, folliculitis, and eventual cystic dilatation [10]. Apocrine glands may discharge into the superficial section of pilosebaceous duct rather than usually opening directly onto the skin's surface. In such cases, apocrine and sebaceous gland enlargement, irritation, and subsequent bacterial infection may result from superficial follicular blockage [9]. According to specific theories, follicular blockage, folliculitis, and ensuing cystic dilation may represent significant early changes in hidradenitis suppurativa [6,7]. The ability of lithium to promote neutrophil migration and phagocytosis, increase epithelial cell proliferation, or directly cause follicular plugging by affecting follicular keratinocytes is hypothesized to be the underlying cause (as in acne) [11,12]. Toll-like receptor (TLR) activation and downstream of TLR are also modulated by lithium [5]. In contrast, recent studies have examined the potential role of modulating TLR activity in the inflammatory pathophysiology of acne inversa. Lithium-induced acne inversa may also result through neutrophilic chemotaxis1 and their degranulation, which set off an inflammatory cascade (as in psoriasis) [6].

According to reports, 3% to 34% of people using lithium therapy experience cutaneous adverse effects [13]. Lithium tablets' inactive components, such as dyes and fillers, could cause an unfavorable reaction, and the female population may be more prone than men to developing adverse effects [9,14]. Management of lithium-induced dermatologic conditions may be resistant to conventional treatment, and discontinuation or dose reduction of lithium can be advantageous sometimes, as in our case. Excellent anti-acne skin care can also be beneficial in fighting breakouts while taking lithium. Cleansing products, including salicylic acid, can reduce swelling and unclog pores [13,15].

## Conclusions

Multiple cutaneous lesions are more common in lithium patients. The lithium dosage and the therapeutic range of serum lithium levels are closely correlated with the frequency of cutaneous adverse effects. Additional research is required to assess the prevalence of acne inversa in patients receiving lithium therapy and the temporal relationship between the start of lithium therapy and the course of acne inversa occurrence. Before starting lithium treatment, the clinician should educate the patient to reduce attrition. Since dermatological care cannot treat lithium-induced skin problems, the doctor needs to be concerned about switching to alternative mood stabilizers.
